# End-tidal carbon dioxide monitoring during bag valve ventilation: the use of a new portable device

**DOI:** 10.1186/1757-7241-18-49

**Published:** 2010-09-14

**Authors:** Veronica Lindström, Christer H Svensen, Patrik Meissl, Birgitta Tureson, Maaret Castrén

**Affiliations:** 1 Karolinska Institutet, Department of Clinical Science and Education, Södersjukhuset, Stockholm, Sweden; 2 Karolinska Institutet, Department of Clinical Science and Education, Section of Anesthesiology and Intensive Care, Södersjukhuset, Stockholm, Sweden; 3 Karolinska Institutet, Department of Clinical Science and Education, Section of Emergency Medicine, Södersjukhuset, Stockholm, Sweden

## Abstract

**Background:**

For healthcare providers in the prehospital setting, bag-valve mask (BVM) ventilation could be as efficacious and safe as endotracheal intubation. To facilitate the evaluation of efficacious ventilation, capnographs have been further developed into small and convenient devices able to provide end- tidal carbon dioxide (ETCO_2_). The aim of this study was to investigate whether a new portable device (EMMA™) attached to a ventilation mask would provide ETCO_2 _values accurate enough to confirm proper BVM ventilation.

**Methods:**

A prospective observational trial was conducted in a single level-2 centre. Twenty-two patients under general anaesthesia were manually ventilated. ETCO_2 _was measured every five minutes with the study device and venous PCO_2 _(PvCO_2_) was simultaneously measured for comparison. Bland- Altman plots were used to compare ETCO_2, _and PvCO_2_.

**Results:**

The patients were all hemodynamically and respiratory stable during anaesthesia. End-tidal carbon dioxide values were corresponding to venous gases during BVM ventilation under optimal conditions. The bias, the mean of the differences between the two methods (device versus venous blood gases), for time points 1-4 ranges from -1.37 to -1.62.

**Conclusion:**

The portable device, EMMA™ is suitable for determining carbon dioxide in expired air (kPa) as compared to simultaneous samples of PvCO_2_. It could therefore, be a supportive tool to asses the BVM ventilation in the demanding prehospital and emergency setting.

## Background

In a prehospital setting, it is necessary that airway management is easily attempted and maintained [1]. Endotracheal intubation (ETI) is regarded as the gold standard for airway management in advanced life support but the procedure requires training and experience [2-4]. Prehospital ETI does neither increase survival rate nor neurologic outcome in trauma patients [5]. Therefore, bag-valve mask (BVM) ventilation should be the preferred technique as it is as efficacious and safe, particularly if healthcare providers are unexperienced [1,3,4,6]. On the other hand, it is most important to provide successful airway management using BVM [7]. Guidelines from the European Resuscitation Council (ERC) describes that all health care providers should be traind to use BVM for ventilation during cardiopulmonary resuscitation [8]. BVM, however, is dependent on provider technique and to facilitate the evaluation of this it could be beneficial to use a small capnography device (EMMA™).

The aim of this study was to investigate whether a new portable device attached to a ventilation mask can give end- tidal carbon dioxide (ETCO_2_) values corresponding to carbon dioxide measurements from venous blood gases (PvCO_2_).

## Methods

This was a prospective observational study. The study was approved by the Ethical Board of the Stockholm County, Stockholm, Sweden (2009/652-31/3). Twenty-two women undergoing breast surgery were included after they had given their written informed consent to participate. The surgeries consisted of mastectomies with or without evacuation of the axilla as well as other breast reconstruction work. The patient median age was 56 years (range 40-77) and they were all classified as ASA I or II according to the American Society of Anesthesiologists. The procedure was as follows: The patients were brought to the operating room where venous cannulas for sampling of blood were inserted antecubitally. They were monitored by ECG, pulsoxymetry, non invasive blood pressure (AISYS, Datex Ohmeda, WI, USA) and capnography built with mainstream technology (EMMA™ Emergency Capnometer, PHASEIN AB, Danderyd, Sweden) attached to a bag-valve apparatus. Before the patients were anaesthetized, vital signs were recorded and the patients were all hemodynamic and respiratory stabile prior to anaesthesia. The values are shown in Table 1.

**Table 1:** Vital signs prior anaesthesia

	Range	Median
**Pulse rate **beats/min	54-108	73
MAP* mmHg	63-122	97
**Respiratory rate **breaths/min	8-16	12
**P-Saturation %**	94-100	98

* MAP = Mean Arterial Pressure

**Figure 1:**
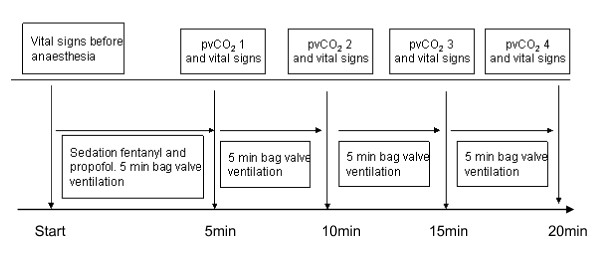
**Flowchart for the study procedure**.

The patients were anaesthetized with a dose of fentanyl (1.4 micrograms/kg) followed by propofol for induction (2 mg/kg). After induction, the patients were put on an infusion of propofol (0.1-0.2 milligrams/kg/min) according to hospital practice. To establish the level of adequate anaesthesia, a clinical assessment (unconsciousness, cessation of spontaneous ventilation, absence of eye lash and bulb reflexes) was made by the attending anaesthesiologist to evaluate that the patient was properly anesthetized. The patient was ventilated by bag-valve mask during the whole study period. The total time of bag-valve ventilation for evaluation of the new device lasted at least 20 minutes. After the study period ended, a laryngeal mask was inserted and the breast surgery was performed. The same anaesthesiologist was the sole provider of bag-valve ventilation for all twenty-two patients. Every 5 minutes during the study period, sampling occurred for PvCO_2 _readings together with simultaneous readings from the EMMA™ device (time points 1-4, with 5 minutes in between). The blood samples for venous blood gases and vital signs were collected by the same nurse. All blood gases (PvCO_2_) were analysed at a nearby analyzer (Radiometer ABL 520, Copenhagen). See flowchart for the study procedure (Fig 1).

### Statistics

Bland-Altman plots were used to investigate the differences between the EMMA™ device and venous blood gases at time points 1, 2, 3 and 4, where most of the differences between the two methods (95%) were expected to lie within the limits of agreement. The assumption of normality was investigated with QQ-plots and the Shapiro-Wilk W test. The Bland-Altman plots were performed using R version 2.9.2. All descriptive statistics used to illustrate the hemodynamic profile of the women undergoing breast surgery during bag-valve ventilation analysis was carried out using Microsoft Excel.

## Results

**Figure 2:**
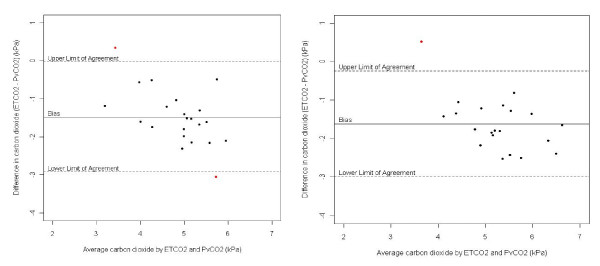
**Bland-Altman plots**.

There were no missing data concerning measurement of vital signs and ETCO_2 _during the study. Regarding to PvCO_2 _there were three (3) missing observations in blood sample two, three and four for the same patient. The patients were all hemodynamic and respiratory stable during anaesthesia. The hemodynamic and respiratory values are shown in Table 2. Bland-Altman plots are displayed for time points 1 and 3 (Fig 2). The bias, limits of agreement (LoA), and the associated confidence intervals are displayed in Table 3. A violation to the distributional assumption of normality was detected for time point 2. Due to interpretability and comparability over the time points no transformation was however performed and therefore the results should be considered with some caution for this time point. The bias, the mean of the differences between the two methods (device versus venous blood gases), for time points 1-4 ranges from -1.37 to -1.62. The associated limits of agreement were similar for all time points and ranged from -3.17 (lower) to 0.25 (higher).

**Table 2:** Hemodynamic and respiratory values during study

Time point = 1	Range	Median
**Pulse **(beats/min)	41-97	61
MAP* mmHg)	63-122	65
**Respiratory Rate**		
breaths/min	8-12	9
**Tidal Volume**	200-703	476
ml/breath		

**t = 2**		
**Pulse rate **(beats/min)	50-103	64
**MAP **(mmHg)	56-80	66
**Respiratory Rate**		
breaths/min	5-12	8
**Tidal Volume**		
ml/breath	135-674	517

**t = 3**		
**Pulse rate **(beats/min)	47-95	62
**MAP **(mmHg)	56-92	62
**Respiratory Rate**		
breaths/min	5-12	8
**Tidal Volume**		
ml/breath	140-667	501

**t = 4**		
**Pulse rate **(beats/min)	43-94	60
**MAP **(mm Hg)	52-74	62
**Respiratory Rate**		
breaths/min	6-15	8
**Tidal Volume** ml/breath	140-657	525

* MAP = Mean Arterial Pressure

**Table 3:** Bias, limits of agreement and the associated confidence intervals

Minutes Bag valve Ventilation	Time point in article	Bias (Mean difference)	95% confidence interval for bias	Limits of agreement (Lower LoA, Upper LoA)	95% confidence interval for LoA (CI Lower LoA; CI Upper LoA)
5	1	-1.48	-1.80, -1.15	-2.92, -0.03	-3.56, -2.28; -0.68, 0.61
10	2	-1.37	-1.66, -1.08	-2.64, -0.09	3.24, -2.04; -0.70, 0.51
15	3	-1.62	-1.93, -1.31	-2.99, -0.24	-3.62, -2.37; -0.87, 0.38
20	4	-1.46	-1.85, -1.08	-3.17, 0.25	-3.88, -2.47; -0.46, 0.95

## Discussion

The aim of this study was to compare the efficacy of a new portable device, EMMA™, for measuring carbon dioxide in expired air compared to carbon dioxide levels in venous blood. The point was to see whether this device could be used as an auxiliary tool for evaluating the accuracy of bag-valve mask ventilation. The main conclusion is that when patients are well under anaesthesia, are hemodynamically stable and adequately ventilated by a trained provider, the device gives acceptable values for exhaled carbon dioxide as compared to venous blood gases. However, our results may not necessarily be transferable to less experienced BVM provider and patients in the prehospital settings. Further studies should include patients and health care providers from the prehospital setting. In an emergency setting, patients are not normally well monitored. Furthermore, many untrained personnel are involved and adequate airway management is sometimes difficult to evaluate. Conventionally, for unconscious patients, ETI is regarded as the gold standard for airway management in ALS, even if the airway management can be easily maintained [1]. However, several studies point to difficulties in using ETI in prehospital settings [3,4,6]. Furthermore, prehospital ETI does not appear to have benefits over BVM ventilation and it does not seem to improve neither survival nor neurologic outcome [5]. Particularly, there are disadvantages using ETI in prehospital settings when the procedure is performed by less experienced paramedics or when the tube cannot be inserted due to the lack of experience from necessary anaesthetic drugs. BVM ventilation is the basic technique for all health care providers [1] and guidelines from ERC states that all health care providers should be familiar with the BVM for ventilation during cardiopulmonary resuscitation [8].

There is an increasing interest for the use of end-tidal carbon dioxide measurement in the emergency care and previous studies have for i.e. described how nasal entidal carbon dioxide measurement could assess patients' acute respiratory problems in prehospital settings [9,10]. In this study we evaluated the EMMA™ device during BVM ventilation under ideal conditions with a trained provider and healthy patients were included.

Capnography is a non-invasive infrared spectroscopy technology for continuous measurement of carbon dioxide (CO_2_) content throughout the respiratory cycle. When capnograms are used to evaluate the end-tidal concentration of carbon dioxide it must be interpreted in conjunction with other clinical findings such as the work of breathing, CO_2 _transport and elimination as well as changes in cardiac output during volume resuscitation [11]. Normally, when the partial pressure of carbon dioxide is measured invasively there is a slight discrepancy between blood values and expired carbon dioxide due to dead space of the lung and bronchial tree. This gradient is low, usually around 0.66 kPa at a lower ETCO_2 _level. This gradient, however, could increase due to patient aging [12]. This was not adjusted for in this study. The results in this study underlines that when the patients are comfortably anaesthetized there is an acceptable agreement between ETCO_2 _values by the device and simultaneously collected PvCO_2 _blood samples. The Bland-Altman plots (Fig 2) show agreement between ETCO_2 _and PvCO_2 _within 2 SD. The limits of agreement are wide, reflecting the large variation, but considered clinically acceptable in view of the normal difficulties of providing an adequate airway by using BVM and also the spread of different ages of the patients. The strength in the study is that the same experienced anaesthesiologist was the sole provider of ventilation for all the patients. This can also be a limitation as he is able to influence the measurement from the device during BVM. The study did not start until the patients were fully anaesthetized and hemodynamically stable. The patients chosen were all ASA I and II and therefore easily maintained. A weakness could be the difficulty of keeping an adequate airway by BVM. This is highly dependable on the provider skill and technique. Furthermore, we used venous blood gases for simplicity and the lack of an arterial line. Mixed venous blood gases reflect desaturated blood which should more easily attract CO_2 _due to the Haldane effect [11,13]. However, a recent study illuminates that peripheral venous blood correlates reasonably well with arterial values, at least for ph, bicarbonate and PCO_2 _[14].

## Conclusions

We conclude that, the portable device, EMMA™ is suitable for determining carbon dioxide in expired air (kPa) as compared to simultaneous samples of PvCO_2_. It could therefore, when the patient has an inadequate respiration, be a supportive tool to assess the BVM ventilation provided there is adequate circulation.

## Competing interests

The authors declare that they have no competing interests.

## Authors' contributions

VL, CS and MC conceived and designed the study. VL and PM collected data. Analyses were made by VL, CS, MC and all authors contributed substantially to the manuscript. All authors have read and approved the final manuscript.
